# Biomineralization by Extremely Halophilic and Metal-Tolerant Community Members from a Sulfate-Dominated Metal-Rich Environment

**DOI:** 10.3390/microorganisms10010079

**Published:** 2021-12-31

**Authors:** Marie Harpke, Sebastian Pietschmann, Flávio Silva Costa, Clara Gansert, Falko Langenhorst, Erika Kothe

**Affiliations:** 1 Institute of Microbiology, Friedrich Schiller University Jena, Neugasse 25, D-07743 Jena, Germany; marie.harpke@uni-jena.de (M.H.); sebastian.pietschmann@uni-jena.de (S.P.); flavio.silvacosta@uni-jena.de (F.S.C.); clara.gansert@uni-jena.de (C.G.); 2 Institute of Geosciences, Friedrich Schiller University Jena, Carl-Zeiss-Promenade 10, D-07745 Jena, Germany; falko.langenhorst@uni-jena.de

**Keywords:** sulfate-rich environment, halotolerance, metal tolerance, community adaptation, biomineralization, strontium struvite, cesium struvite

## Abstract

The adaptation to adverse environmental conditions can lead to adapted microbial communities that may be screened for mechanisms involved in halophily and, in this case, metal tolerance. At a former uranium mining and milling site in Seelingstädt, Germany, microbial communities from surface waters and sediment soils were screened for isolates surviving high salt and metal concentrations. The high salt contents consisted mainly of chloride and sulfate, both in soil and riverbed sediment samples, accompanied by high metal loads with presence of cesium and strontium. The community structure was dominated by Chloroflexi, Proteobacteria and Acidobacteriota, while only at the highest contaminations did Firmicutes and Desulfobacterota reach appreciable percentages in the DNA-based community analysis. The extreme conditions providing high stress were mirrored by low numbers of cultivable strains. Thirty-four extremely halotolerant bacteria (23 *Bacillus* sp. and another 4 Bacillales, 5 Actinobacteria, and 1 Gamma-Proteobacterium) surviving 25 to 100 mM SrCl_2_, CsCl, and Cs_2_SO_4_ were further analyzed. Mineral formation of strontium- or cesium-struvite could be observed, reducing bioavailability and thereby constituting the dominant metal and salt resistance strategy in this environment.

## 1. Introduction

Environments with high salt concentrations such as brines, salty soils, or sediments drive adaptation of the microbial community present at such sites [1,2,3,4]. Specific contaminants include not only NaCl, but specifically due to mining operations of sulfidic ores, high sulfate concentrations may lead to halophilic communities establishing on such sites [5,6,7]. Usually, high salinity is observed concomitantly with high pH [1,8,9]. However, former mining sites are associated with circum-neutral or even acidic pH, the latter promoting metal leaching. The co-occurrence of metal ions poses another stress on the microbial community, which results in co-tolerance [10,11].

An example of such a site where acid mine drainage with high metal and sulfate loads at circum-neutral pH is the former uranium mining and milling site near Seelingstädt, Germany, where remediation activities started in 1990 including dry in situ stabilization of the tailings, re-vegetation, and the treatment of seepage waters [12,13]. A total of about 110,000 t U were processed from 1960 to 1990 [14]. The valley between two tailings has received sulfate–chloride rich tailings seepage with likely high metal contents for over 30 years. In the 1960s, one tailings dam failed, releasing tailings material that was subsequently removed [15].

In addition to high sulfate concentrations, metal ions present a specific challenge for microorganisms. While resistance against Ni, Zn, Cu, or Cd has been investigated in many studies, metals derived from U decay such as Cs or Sr are underrepresented in such investigations [16,17,18,19]. The high halo-metal contamination requires microorganisms to keep an osmotic balance [20], as well as coping with high metal loads. The mechanisms employed by microorganisms to withstand saline conditions are generally classified into salt-in and salt-out strategies [21,22,23]. Salt-out halotolerance is dependent on intracellular compatible solutes such as sugars, amino acids, glycine betaine, trehalose, or ectoine [24,25,26], and decreasing salt concentrations have been found to lead to higher sensitivity towards cadmium, copper, and cobalt [11,27].

To compare microbiomes present at contaminated sites, pristine soils can serve as a basis. Acidobacteria, and in some lower counts, Verrucomicrobia and Bacteroidetes represent the highest abundances in soils world-over [28]. The fungal community in soil is usually dominated by Basidiomycota and Ascomycota. Adaptation to co-occurring salt–metal stress requires specific mechanisms [29]. Microorganisms are able to accumulate heavy metals through biosorption and uptake; another potential way to prevent influx of salt and metals can be seen with biomineral formation in the vicinity of the cells, since the reduced bioavailability will reduce the selection pressure [15,30]. With this line of thought, the formation of extracellular biominerals would constitute a selective advantage, and at the same time, provide a benefit not only for the producer, but would also reduce environmental stress to any other community member. The co-contaminated environment at a former uranium mining site was therefore investigated to identify specific communities and adaptation strategies in community members to examine the hypothesis of enhanced biomineralization being present specifically at such sites.

The community structure along a flow path receiving sulfate- and metal-rich seepage waters that, in the past, have exceeded today’s input was chosen to test our hypothesis. We expected to find a high diversity, but low richness along the contamination gradient. To assess highly halotolerance or halophilic strains, soil, sediment, and water were used to isolate microorganisms. Microbiome analysis on soil and sediment samples were performed to test whether environmental factors in combination with certain fungal species would determine the bacterial community. The isolated bacteria were investigated for their salt–metal tolerance ability and to identify mechanisms including biomineralization capacity that would allow them to survive or even thrive in the given environment of the former uranium mining site.

## 2. Materials and Methods

### 2.1. Sampling Sites and Sediment Analyses

Soil, sediment, and water samples were taken at sites A–F to reflect an increasing contamination gradient obtained by hydrogeochemical mapping (Supplementary Figure S1, created using QGIS v3.22.1) in a natural wetland between two tailings of the former uranium mining and milling site near Seelingstädt, Thuringia, Germany. At site A (303553 E, 5627471 N, 795 µS/cm), water as well as creek sediments (below the water surface) were taken from the creek bed and creek, upstream of the influence of contaminants from the two tailings. At site B (303306 E, 5626999 N, 501–750 µS/cm), soil from an upstream pasture was analyzed. Here, the soil sample was very fresh. Site C (303421 E, 5627443 N, 1684 µS/cm) represented soil from a reed swamp with dark brown, organic-rich soil. The sample was water-logged. From site D (303475 E, 5627371 N, 9670 µS/cm) at the tailings inlet, both water and sediments (taken below the water table) were examined. Site E (303190 E, 5627009 N, 100,900 µS/cm) allowed water sampling from dripping water as well as soil sample analysis from a red-brownish, gleyic, and water-logged soil. At site F (303274 E, 5627001 N, 9700 µS/cm), which was located downstream from the visible water entry into the creek, sediment samples (below the water surface) as well as water samples were collected from the creek bed.

To be able to compare time series in addition to the special distribution, several sampling campaigns were conducted. Soil/sediment samples were taken during spring 2018, winter 2018/2019, and autumn 2019 after removing 5 cm of the soil profile, using autoclaved spoons. Approximately 10 g was transferred into sterile 100 mL sample vessels (Fisher Scientific, Schwerte, Germany), transported at 4 °C and processed on the same day. Water samples were obtained in spring 2018 (site E), or autumn 2019 (sites A, D, F).

For chemical analyses, the sampled soil and sediments were dried for one week at 60 °C, crushed in a porcelain mortar and sieved to <2 mm. After sequential extraction [31], bioavailable element fractions 1 (mobile) and 2 (easily mobilized) were measured in three replicates by ICP-OES (simultaneous radial ICP-OES spectrometer 725ES, Agilent, Waldbronn, Germany) and ICP-MS (quadrupole-ICP-MS-spectrometer XSeries II, Thermo Scientific, Bremen, Germany). Water samples were measured for the same element concentrations as well as for elements and ions determining water types by ICP-MS, ICP-OES, and ion chromatography (Integrion-RFIC, Thermo Fisher Scientific, Waltham, MA, USA; conductivity detector Integrion CD with Supressor ADRS 500, and UV/VIS detector VWD-IC, Thermo Fisher Scientific, Waltham, MA, USA). Anion separation columns IonPac AS18-Fast-4 µm with precolumn and IonPac AS11-HC 4 µm with precolumn (Thermo Fisher Scientific, Waltham, MA, USA) and a KOH gradient as eluent were applied. An integrated automated sample preparation removed alkaline, alkaline earth, and transition elements as well as organic impurities (InGuard Na/HRP and enrichment column IonPac UTAC-LP2, Thermo Fisher Scientific, Waltham, MA, USA).

### 2.2. Characterization of Isolates

The fresh soil/sediment sample was suspended (1:10) in 0.9% NaCl and dilutions plated in triplicate onto the isolation media StdI (Roth, Karlsruhe, Germany) with or without NaCl (10% or 20%) added, soil extract minimal medium [32], MEA medium [33], or a low nutrient groundwater medium, R2A, for riverbed sediment samples [34]. After at least one week at 28 °C for soil samples or 10 °C for riverbed sediment samples, single colonies were identified and pure cultures obtained for further analysis. Water samples were plated on R2A medium and cultivated at 10 °C for at least 2 weeks to obtain pure cultures.

Identification of bacterial isolates was performed by 16 S rDNA sequencing from genomic DNA (Chelex 100, Bio-Rad, Hercules, CA, USA) after PCR amplification (2.5 µL primers 27f: 5′-AGAGTTTGATCCTGGCTCAG-3′ and 1492r: 5′-ACGGCTACCTTGTTACGACTT-3′) [35]. PCR was performed with 2.5 μL dNTPs (Jena Bioscience, Jena, Germany), 0.2 μL DreamTaq and 10 µL Taq-buffer (Thermo Fisher Scientific, Waltham, MA, USA), 30.3 µL sterile nuclease-free water (Carl Roth, Karlsruhe, Germany) and 1 μL template with 5 min denaturation at 95 °C, 30 cycles for amplification (30 s denaturation, 95 °C; 45 s, 60 °C; 90 s, 72 °C), followed by final elongation for 10 min at 72 °C using a Thermocycler T3 (Biometra, Göttingen, Germany). For fungal isolates, DNA extraction [36] was followed by ITS amplification (primers ITS1: 5′-TCCGTAGGTGAACCTGCGG-3’ and ITS4: 5′-TCCTCCGCTTATTGATATGC-3′) [37] with 5 µL primers ITS1/ITS4, 1 µL dNTP’s, 0.25 µL DreamTaq, 5 µL Taq-Puffer 10×, 32.75 µL sterile nuclease-free water, and 1 µL template with 34 cycles following the same protocol. For negative control, nuclease free water was used instead of template. Products were checked by gel electrophoresis using 1% *w/v* agarose, stained with ethidium bromide, documented (Herolab Image Doc, Herolab, Wiesloch, Germany) and sequenced (GATC, Konstanz, Germany, or Starseq, Mainz, Germany). Sequence assembly was carried out via BioEdit Sequence Alignment Editor. The assembled sequences were checked for chimeras by UCHIME [38]. Taxonomy assignment and phylogenetic tree reconstruction of the resulting sequences was performed with R based on SILVA v138 or UNITE v8.3 [39,40,41].

Physiological tests included tolerance to high salt stress (StdI containing 10, 20, or 30% NaCl or 10, 14, or 17% Na_2_SO_4_) in 96-well plates (Greiner Bio-One, Kremsmünster, Austria) for 4 weeks at 28 °C with OD measurement (VERSAmax tunable microplate reader, Molecular Devices, Silicon Valley, CA, USA) after 3, 7, 14, 21, and 28 days. Metal tolerance was tested in 24-well plates (Greiner Bio-One, Kremsmünster, Austria) with 2 weeks of growth at 28 °C (StdI containing CsCl, Cs_2_SO_4_, and SrCl_2_ at 25, 50, or 100 mM). Growth was confirmed microscopically (VHX-970F, Keyence Corporation, Neu-Isenburg, Germany).

### 2.3. Microbiome Analyses

Genomic DNA was extracted from soil and sediment samples using the DNeasy PowerSoil Kit (Qiagen, Hilden, Germany). The 16S rDNA was amplified using the universal primer set 341F (5′-CTGNCAGCMGCCGCGGTAA-3′) and 806bR (5′-GGACTACNVGGGTWTCTAAT-3′) and ITS1/ITS2 [37]. The 16S rRNA gene amplicon library was sequenced with 2 × 250 bp reads and the ITS amplicon library with 2 × 300 bp reads on the Illumina MiSeq platform (StarSEQ, Mainz, Germany). After trimming, demultiplexing, denoising, sequence merging, ASV resolving, and chimera removal, analysis and phylogenetic tree reconstruction were performed as described above adding correlation analyses tools available with R. All sequences are available at NCBI GenBank with the accession numbers OL679468-OL679470, OL678420-OL678455, OL679704-OL679837, OL681894-OL681895, and OL685263-OL685271 and NCBI Sequence Read Archive under the Bioproject PRJNA784578.

### 2.4. Biomineral Characterization

Mineralization was scored microscopically (Keyence, Neu-Isenburg, Germany). Control experiments were performed using autoclaved dead biomass or sterile filtered (0.2 µm) culture supernatant.

Mineral identification and characterization were performed by Raman spectroscopy and analytical transmission electron microscopy (ATEM).

The Raman spectra were taken with a WITec (Ulm, Germany) alpha300 M +micro-Raman spectrometer, using a 531,95 nm emission laser. Two gratings with 600 and 1800 grooves were used to measure in the wavenumber ranges 70–1270 cm^−1^ and 230–3730 cm^−1^. To avoid damage by the laser beam we reduced the laser power to moderate values ≤5 Watt. As a drawback we had to prolong the acquisition times up to 2 min.

ATEM was carried out to determine the structural and chemical characteristics of the biominerals. For this purpose, we used a 200 kV FEI Tecnai G2 FEG TEM, which is equipped with an Oxford 80 mm^2^ energy-dispersive silicon drift detector (SDD). In the course of the investigations, it turned out that the biominerals immediate start become amorphous under high vacuum conditions such that selected area electron diffraction was not possible. Despite this, we could however determine the chemical compositions of biominerals by energy-dispersive X-ray spectroscopy.

## 3. Results

### 3.1. Halophilic Conditions with Sulfate-Rich Waters and Metal-Rich Sediments and Soils Characterized the Region

The hydrogeochemical analysis identified increasing conductivities for sampling sites A–F (compare Supplementary Figure S1). At the same sites, metal analyses were performed to evaluate salt versus metal contamination (Supplementary Table S1). As expected from the hydrogeology mapping, site E showed higher concentrations, while metal loads at sites A and B were lowest. While generally, metals including Cu, Co, Fe, Ni, Pb, and Zn showed low concentrations, Fe, and specifically Mn (up to 1595 µg/g) loads in the water were high pointing to acid mine-drainage influence. Sr (7–24 µg/g) reached higher concentrations differing between sampling points and fluctuating with time. The elements Cs and Sr were higher in water than in soil/sediment samples and were chosen for subsequent analyses with respect to an effect on community structure.

Water chemistry could be determined to be dominated by sulfate with 5535 mg/L, followed by high concentrations of chloride with up to 1037 mg/L (see Supplementary Table S1). From these combined values we expected halotolerant adaptation of 5–10% salts.

### 3.2. Microbiomes Were Dominated by Proteobacteria, Chloroflexi, and Acidobacteriota

**Figure 1:**
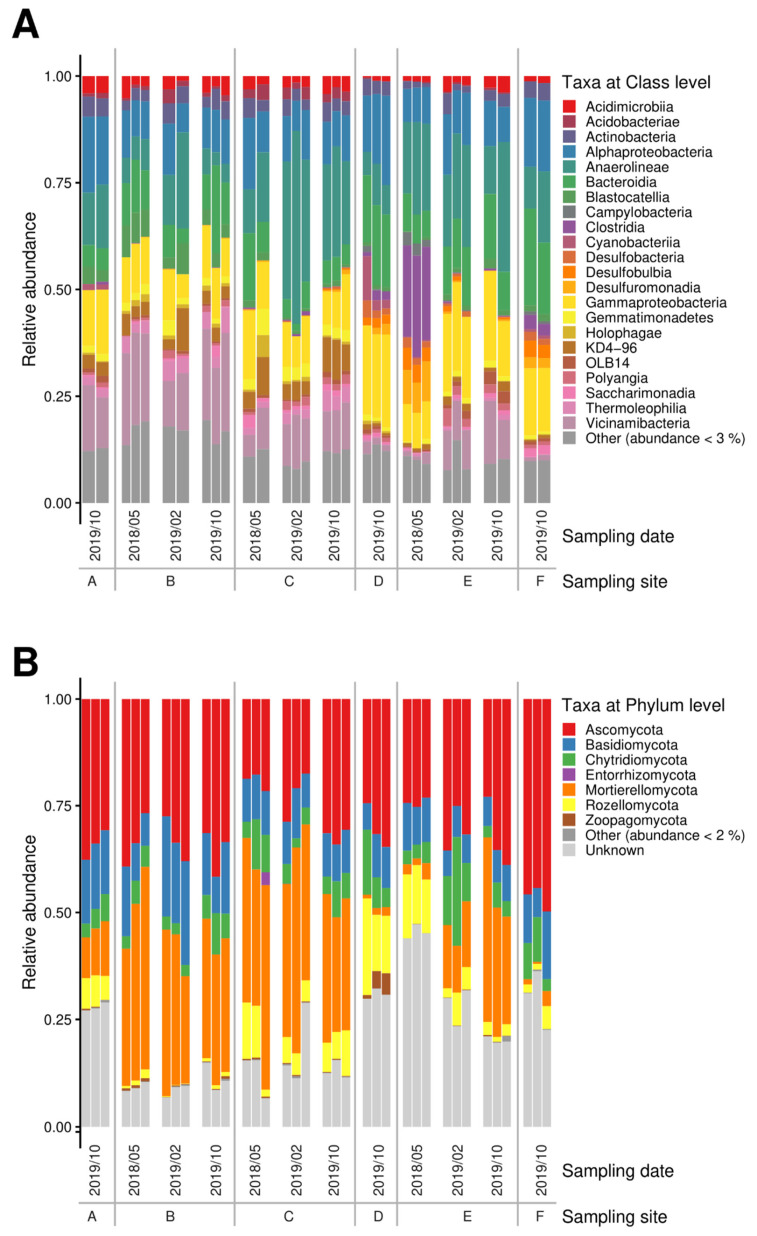
Bacterial (**A**) and fungal (**B**) microbiomes along a contamination gradient from sites A to E (compare Supplementary Figure S1).

The 16S microbiome analyses showed a strong selection for bacterial phyla, with good accordance within the replicates (Figure 1A) as well as stability over time series at each sampling site, except for site E, where the first sampling revealed a marked difference to the other readings. This result, however, confirms the measured higher contamination only seen at that time.

On the phylum level, Proteobacteria, Chloroflexi, and Acidobacteriota dominated, followed by Actinobacteriota and Bacteroidota. Firmicutes, Desulfobacterota as well as Gemmatimonadota were present, albeit at lower read counts. The soil samples taken at different time-points from site E that featured the highest metal loads differed in the microbiome structure with a shift towards Firmicutes and Desulfobacterota, and with Campilobacterota only being visible in these samples. Verrucomicrobiota, Latescibacterota, and Methylomirabilota seemed vulnerable and were found only in samples that had revealed the lowest contamination (compare Supplementary Table S1). In sediment samples, numbers of the dominating phyla were slightly reduced to accommodate Bacteroidota among the highly represented phyla, as well as members of the phylum Patescibacteria with more than 2% read counts in these sediment samples. Cyanobacteria were found in the sediment bacterial community with high representation in the salt-rich sample of site D. At class level, high abundance of Anaerolineae (Chloroflexi) as well as Gammaproteobacteria was recorded in all samples, while Vicinamibacteria and Holophagae (both Acidobacteriota) and KD4-96 (Chloroflexi) were only missing in the most halophilic environment, where instead Clostridia (Firmicutes) became more abundant (see Figure 1). The microbiomes at the site showed high prevalence of Anaerolineae and other Chloroflexi, Vicinamibacteria, and Holophagae (Acidobacteriota) not usually prominently represented in less salt- and metal-rich soils.

The α-diversity was found with high diversity and dominance (Supplementary Figure S2). Again, the changes over time were visible for site E. Using Shannon index, the highest diversities (scoring up to 6) were observed for site B, D, and F, while Simpson index close to 1 revealed high dominance and Chao1 (with indices to over 800) a high richness and abundance of rare groups.

### 3.3. The Fungal Microbiomes Differed According to Moisture

The fungal groups in soil were dominated by Ascomycota, followed by the Mortierellomycota (Figure 1B). Chytridiomycota could be observed in all samples, being most prominent in the soil from site E after a decrease in metal concentrations. Another interesting finding was that the highest contaminated samples of site E contained a specifically high abundance of sequences that could not be identified from databases (‘unknown’).

In the swamp sediment samples, Mortierellomycota were less prominent, while Basidiomycota could be detected, and at one specific site, D, Rozellomycota represented the second most abundant phylum observed. Zoopagomycota were only observed in the high salt, low heavy metal containing sample of site D. With respect to a specific, potentially adapted mycobiome, we found that in contrast to uncontaminated soils, Basidiomycota were found in lower read counts. Instead, Mortierellomycota were found in appreciable numbers. At class level, Agaricomycetes and Tremellomycetes were present in all samples, while Taphrinomycetes were enriched in sediment samples at site D. The Ascomycota were represented by Dothideomycetes (and Sordariomycetes for sediment samples). Rhizophydiomycetes (Chytridiomycota) were found in most the samples, while Enthorrhizomycetes (Entorrhizomycota) and Laboulbeniomycetes (Ascomycota) could only been observed in samples of site B at low contamination, while Olpidiomycetes (Olpidiomycota) were associated at site E featuring high salt and metal loads. The class of Zoopagomycetes (Zygomycota sensu lato) were only observed in sediment samples from site D.

Again, high diversity and high dominance were seen with α-diversity indices (compare Supplementary Figure S3). A lower diversity and richness were observed at site C in soil and site F in riverbed sediments, with the respective indices for Shannon up to 5.0 for soil and 5.6 for sediments, Simpson close to 1 and Chao1 to >600 for soil and >900 for sediment samples (with lower values for site F samples).

### 3.4. Which Factors Determined the Bacterial and Fungal Community Structure?

**Figure 2:**
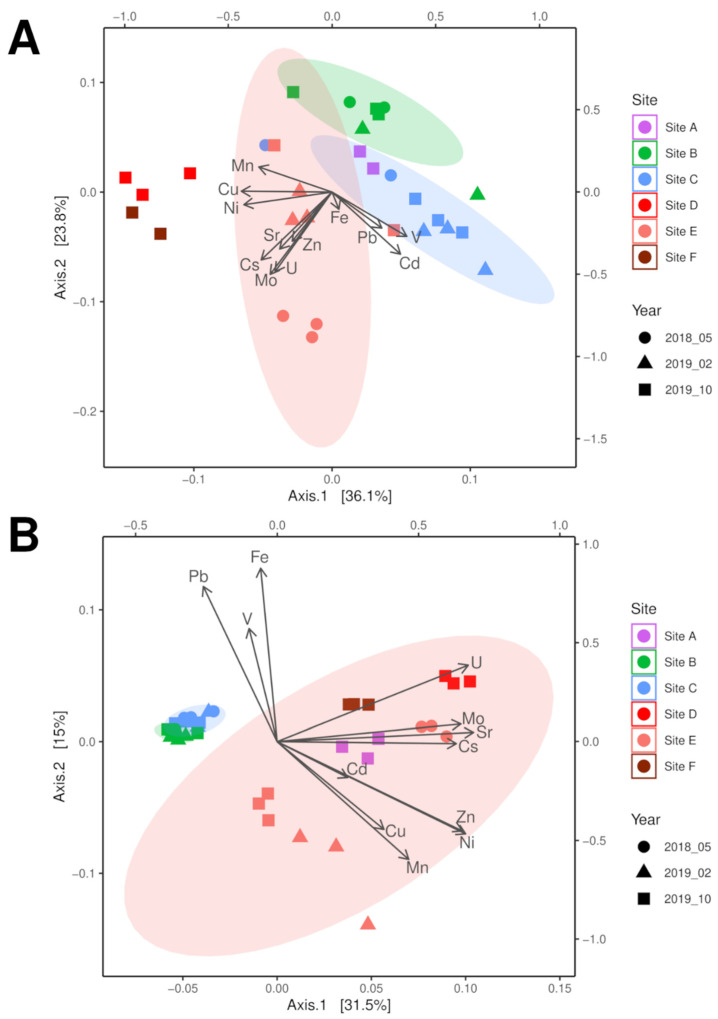
PCoA analysis of bacterial 16S (**A**) and fungal ITS (**B**) microbiome data correlated with physicochemical soil parameters in a contamination gradient from sampling site A through site F (compare Supplementary Table S1).

Principal coordinates analysis (PCoA) of the microbiome sequencing data identified that the three soil sampling points were separated by contamination (highest at site E), which became more similar over time with decreasing contamination at site E (Figure 2A). While the community of sampling point C was mainly described by the presence of the metal ions Fe, Pb, Cd, and V, the highly contaminated sampling site E showed a strong separation due to Cs and Sr, but also correlated well with Zn, Cu, Mn, and Ni. With respect to riverbed sediment samples, a similar separation between low contamination at site A and the other sites located downstream of a tailing water inflow (see Figure 2A) more similar to soil data was found. The two riverbed sediment samples of site D and F were well separated by the higher concentrations of Cu, Ni, and Mn, with higher similarity the microbiome of the highest contaminated soil sample of site E.

The fungal communities were separated between soil and riverbed sediment samples, with the contaminated sampling site E becoming more similar to the soil samples with decreasing contamination (Figure 2B, compare Supplementary Table S1) associated with the bioavailable concentrations of Cs, Cu, Mn, and Mo as well as decreasing moisture. Since all riverbed sediment samples plotted within the confidence interval of site E, but metal loads differed, moisture of the sample seemed to be the determining factor for the fungal community. The soil microbiome of sampling site C was mainly separated by the metals Fe, Pb, and V, as observed with the bacterial analyses. Thus, both fungal and bacterial communities showed adaptation to salt and metal stress. This prompted us to look more specifically for physiological traits that allow for survival in the halo- and metal-rich environment.

### 3.5. Isolation of Halo- and Metallo-Tolerant Strains Yielded Mainly Spore Forming Species

From all samples, different media were inoculated to obtain different strains for further cultivation and physiological testing. The abundance of microbes dropped from soil over sediment to water samples (Supplementary Table S2). At the same time, the highest salt and metal loads at sampling site E led to lower numbers in all compartments as compared to the other sites. This finding confirms the difference observed in microbiome data. The addition of salt to the isolation medium decreased the abundance of cultivable strains, as expected. The use of a medium that should support fungal growth yielded lower bacterial counts, but since no antibiotic was added, still more bacterial as compared to fungal isolates were obtained.

**Figure 3:**
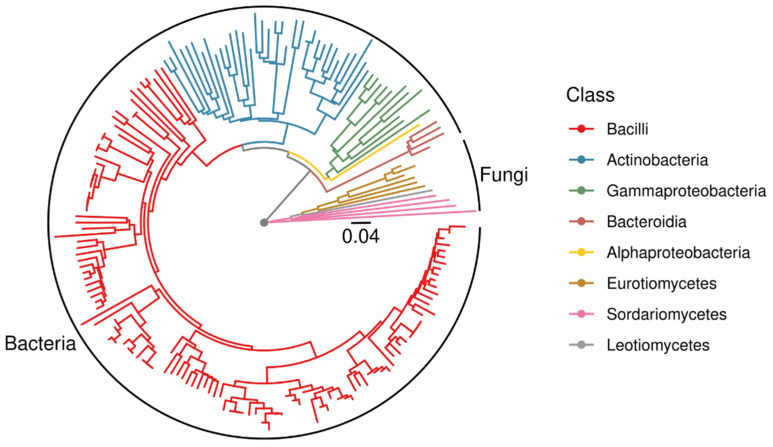
Midpoint-rooted phylogenetic tree of all isolates based on 16S rDNA and ITS sequences.

From the isolation plates, 181 pure cultures were obtained. These covered a wide range of phylogenetic groups. However, in contrast to the microbial community analyses, among the isolates Bacillaceae were highly dominated (Figure 3). Halophilic genera such as *Halobacillus* and *Halomonas* were present, already indicating adaptation to salt-rich environments. Especially from sediment samples, Actinobacteria were enriched in the isolated subpopulation. The fungi were mainly Ascomycota. Hence, endospore- forming Firmicutes and exospore-forming Actinobacteria (with a high diversity of isolated genera) among the bacteria and conidiospore producing Ascomycota from the fungal kingdom were overrepresented by the cultivation approach.

### 3.6. Co-Tolerance towards Salt and the Heavy Metals Cs and Sr

The isolates were tested for halotolerance versus halophilic traits. Mainly halotolerance was scored; 18 out of 136 tested isolates were halophilic with optimal growth only in halophilic conditions, mainly belonging to known halophilic genera such as *Halomonas* (3), *Mycobacterium* (1), and *Dermacoccus* (1), but also encompassing *Bacillus* (3), *Jeotgalibacillus* (2), *Nocardiopsis* (4), *Nesterenkonia* (2), *Dietzia* (1), and *Streptomyces* (1).

In addition, using different Na salts showed most halophilic strains to be more tolerant towards sulfate than to chloride. Most of the halophilic strains revealed tolerance towards <20% NaCl, while they tolerated Na_2_SO_4_ salts up to saturation (Supplementary Table S3). From the isolates, 33 strains were selected for further analysis including the genera *Bacillus* (*n* = 22), *Jeotgalibacillus* (*n* = 2), *Planococcus*, *Staphylococcus*, *Brevibacterium, Dermacoccus, Dietzia, Mycobacterium, Leucobacter, Rhodococcus,* and *Halomonas*. Since the environment was rich both in salt and metals, mainly Cs and Sr, the ability to grow in Cs- or Sr-rich medium was tested. To reveal selection for sulfate versus chloride tolerance, the salts SrCl_2_, Cs_2_SO_4_, and CsCl were tested. All isolated strains could grow with 100 mM metal salt added to the medium. To separate the metal ion tolerance from halotolerance, the Na salts were used. Much higher concentrations in the molar range were tolerated by the vast majority of the tested strains.

A difference was observed between growth with NaCl versus growth with the sulfate salt (Supplementary Table S3). While with NaCl, a drop from 67 to 31% of all isolates being able to grow occurred between 10 and 20% of salt added to the medium, no relevant drop was observed between 10 and 17% of the sulfate salt added to the media. Translated into molarities, the observed resistances translate to 1.7, 3.7 and 5.1 M NaCl and 0.7, 1.4 and 2.1 M Na_2_SO_4_. It can thus be confirmed that the tolerance towards sulfate exceeded the mere osmolarity also reached with the chloride salt. Among the tested genera, many bacilli were specifically salt tolerant or even halophilic (Supplementary Figure S4).

At the highest concentrations, the solubility of the respective salt was reached, leading to crystal formation. A microscopic inspection revealed that minerals mostly occurred around the inoculated bacteria, which indicated biomineralization (Supplementary Table S4). This would lead to lower ionic strength surrounding colonies on agar plates. This may be expected to be a valuable tolerance mechanism, which warranted an investigation of the mineral composition of these biominerals.

### 3.7. Biomineral Formation

**Figure 4:**
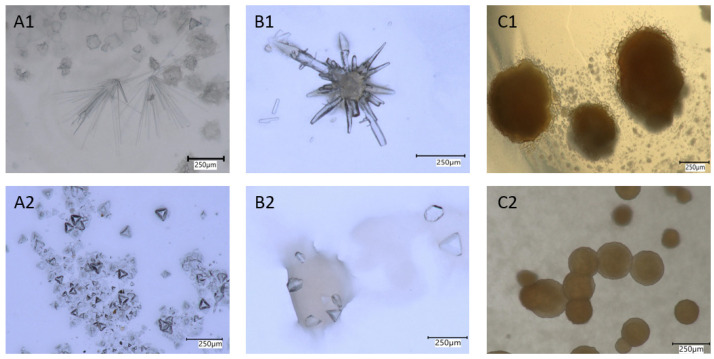
Minerals formed on Cs- (**A**,**B**) and Sr- (**C**) containing media. (**A1**) Needle-like aggregates and botryoidal crystals formed with Cs_2_SO_4,_ (**A2**) tetrahedrons formed with Cs_2_SO_4,_ (**B1**) close-up of needle aggregates and (**B2**) aggregates, formed with CsCl, (**C1**) brown-colored aggregates, and (**C2**) singletons formed with SrCl_2_.

To support a biological mineral formation process, autoclaved biomass as well as cell-free supernatant were incubated. No crystal formation was observed in those controls, supporting the view that an active involvement of the bacteria is required. The formed crystals differed chemically and structurally from those of the salts applied, and developed various habits. Crystals grown on Cs-containing media formed congregations of prismatic crystal habits or display a combination of faces with pyramidal appearance. A prominent, brown botryoidal mineral either in congregations or singletons was observed in experiments with Sr (Figure 4).

To identify the minerals formed, Raman spectra were acquired and the composition of the crystals was measured by energy-dispersive X-ray (EDX) spectroscopy on an analytical TEM. Raman spectra of crystals grown on all Cs-rich media were fully compatible with the biomineral struvite (Supplementary Figure S5). As noted earlier, these crystals turned out to become amorphous rapidly under the high vacuum conditions of TEM such that no diffraction data could be taken. EDX show, however, that the cation dominating in the struvite structure was Cs and thus its simplified formula is CsMg(PO_4_)6H_2_O.

The brownish aggregates grown in Sr-rich media displayed a Raman spectrum with no distinct bands. Subsequent TEM inspection showed that the aggregates were apparently composed of two phases. Evaluation of electron-diffraction patterns taken in the TEM revealed, however, that the diffraction rings were compatible with only one phase possessing the crystal structure of SrCl_2_. According to EDX measurements, the second phase was mainly composed of Sr, P, N, and O. Considering the afore-mentioned instability of struvite under high vacuum and this composition it is likely that the second phase was also struvite with the simplified composition [NH_4_]Sr(PO_4_)∙6H_2_O (Supplementary Figure S6). However, the purity of the crystals was unexpected, as Ca was present in the growth medium.

## 4. Discussion

Microorganisms are known to be good accumulators of several heavy metals and radionuclides as well associated elements such as S and P [31,42]. They play a key role in biogeochemical cycles such as metal and mineral transformations and bioweathering [42,43,44]. They can be applied to bioremediation approaches reducing the impact of high contamination caused by salt or heavy metals including radionuclides [12]. We expected to find highly different, specialized microbiomes in a gradient of contamination with high salt and metal concentrations [1,2,3,4]. The site featured high Cs and Sr concentrations, which were accompanied by Mn and sulfate as well as chloride concentrations at high levels.

We hypothesized that this specifically challenging environment might be reflected in a community of high diversity, but low richness. However, we found an unexpectedly high richness as well as high diversity. High dominance was indicated and the microbiomes showed good separation according to salt and metal loads. The fungal communities were most dependent on soil moisture. The fungal community in soil is usually dominated by Basidiomycota and Ascomycota [45,46]. Fungi are described to be mainly influenced by the plant population of a site as well as water availability and other abiotic factors such as N-richness, carbon content, or soil quality [46,47,48,49]. Mortierellomycota are often found in heavy-metal- and radionuclide-contaminated, disturbed soil and they are known for accumulation of heavy metals such as Cd, Co, Hg, Ni, Zn, and U [6,50,51] Here, we could show Mortierellomycota in high abundances correlated with Sr and Cs gradients in a saline environment. Rozellomycota (Basidiomycota) were found enriched in the most contaminated samples confirming their occurrence in (saline) wetlands, including the vicinity of a uranium mine [52]. In wetlands, Rozellomycota were found to even exceed the portion of Ascomycetes in the microbiome at progressed stages of litter decomposition. This phylum was also observed in heavy-metal-contaminated water samples and paddy soil [53,54,55].

In addition to Campilobacterota, Firmicutes, and Desulfobacterota are often reported from highly contaminated sampling sites, and Desulfobacterota (with Desulfobacteria, Desulfobulbia, and Desulfuromonadia) specifically relate to the high sulfate concentrations found along the gradient. This supports our thesis, that heavy metal/radionuclide contamination changes the microbial diversity and community [29,56,57]. Acidobacteriota, and in some lower counts, Verrucomicrobiota and Bacteroidota were found in accordance with reports from different soils [28]. Chloroflexi are commonly found in saline as well as uranium- and heavy-metal-containing environments [52,58,59] including soils in the vicinity of the Fukushima nuclear power plant [60]. Halotolerant species such as *Brevibacterium* sp., *Halobacillus* sp., and *Halomonas* sp. were isolated from sampling site E.

*Bacillus* sp. feature a well-balanced osmoregulation depending on expression through the sigma factors SigB, SigW, SigM, and SigX. It forms (and can import) the osmolytes proline and glycine betain [61,62,63]. However, tolerance of the maximum soluble salt has not been reported. The isolated non-Bacillales genera *Brevibacterium, Halomonas, Mycobacterium, Rhodococcus, Dermacoccus, Dietzia,* and *Leucobacter* are known to encompass halotolerant species.

## 5. Conclusions

Adverse environmental conditions are prevalent at former mining sites, with usually high sulfate and associated (heavy) metals contamination. Here, we addressed such an environment in a former uranium mining area for adaptation of the microbial communities. Surface waters, creek sediments, and soils were screened for isolates surviving high salt and metal concentrations. High diversity, but low richness along the contamination gradient was observed. Firmicutes and Desulfobacterota were present in higher amounts only at the sites reflecting the highest contamination.

To obtain strains for physiological test relating specifically to the contamination with Cs and Sr found at the site, cultivation was performed including high-salinity media. This led to isolation of extremely halotolerant and halophilic bacteria. Usually, high salinity is observed concomitantly with high pH. However, former mining sites are associated with non-alkaline or even acidic pH, which promotes metal leaching [12]. At circumneutral pH such as the niche investigated, co-occurrence of (sulfate) salt ions and metal ions imposes extra stress on the microorganisms, which results in co-tolerance [10,11,12].

Resistance mechanisms prevalent in a metal-rich environment were checked, and biomineralization of Cs- and Sr-struvite was identified to be present in most of the adapted strains, constituting the dominant metal and salt resistance strategy in this environment. This capacity contributes to survival in the contaminated environment of a former uranium mining site. The reduced bioavailability can be maintained specifically in structured environments such as soil, where diffusion gradients are steeper as compared to water as an environment. This allowed the identified strains to survive and thrive at the extremely high concentrations of 100 mM Sr or Cs.

The formed Cs-struvite and Sr-struvite may be of specific interest for bioremediation approaches when application of bacilli could lead to reduction of bioavailable contents of these potential radionuclides. At such challenging sites, a microbiological amendment may reduce plant-available Cs and Sr concentrations allowing for advanced, microbially aided phytoremediation [64].

## Data Availability

All data are freely available at accessions OL679468-OL679470, OL678420-OL678455, OL679704-OL679837, OL681894-OL681895, and OL685263-OL685271 and NCBI Sequence Read Archive under the Bioproject PRJNA784578 in NCBI genbank.

## Supplementary Materials

The following are available online at https://www.mdpi.com/article/10.3390/microorganisms10010079/s1, Table S1: Element concentration in three independent samples were measured for soil or sediment samples. Table S2: Microbial count in soil/sediment (cfu/g) and water (cfu/mL) samples. Table S3: Salt tolerance of isolates grown on NaCl or Na_2_SO_4_ containing media. Table S4: Minerals formed on different salt containing media and mineral micromorphology per isolate. Figure S1: Map of the sampling area with sites A (lowest influence) through F (highest contamination) along a salinity gradient resulting from seepage waters from two tailings located to the northwest and southeast. Figure S2: Alpha-diversity measurements of the indices Chao1 as estimator for richness, Shannon for diversity, and Simpson for dominance in bacterial microbiomes to compare sampling points over time. Figure S3: Alpha-diversity measurements of the indices Chao1 as estimator for richness, Shannon for diversity, and Simpson for dominance in fungal microbiomes to compare sampling points over time. Figure S4: Distribution of salt tolerance for genera isolated as per growth in salt-rich media. Figure S5: Raman spectra of crystals that precipitated in experiments with CsCl, Cs_2_SO_4_, and SrCl_2_ solutions in comparison to a reference spectrum of struvite. Figure S6: Energy-dispersive X-ray (EDX) emission spectra of crystals that precipitated in experiments with CsCl, Cs_2_SO_4_, and SrCl_2_ solutions.
