# Supplementary material for: Biomineralization by Extremely Halophilic and Metal-Tolerant Community Members from a Sulfate-Dominated Metal-Rich Environment
Source: Microorganisms. 2021 Dec 31;10(1):79. doi: 10.3390/microorganisms10010079 (PMC8780871; doi:10.3390/microorganisms10010079)
Supplement: Supplementary file 1 [file microorganisms-10-00079-s001.zip › microorganisms-1525182-supplementary.pdf]

Supplemental Material

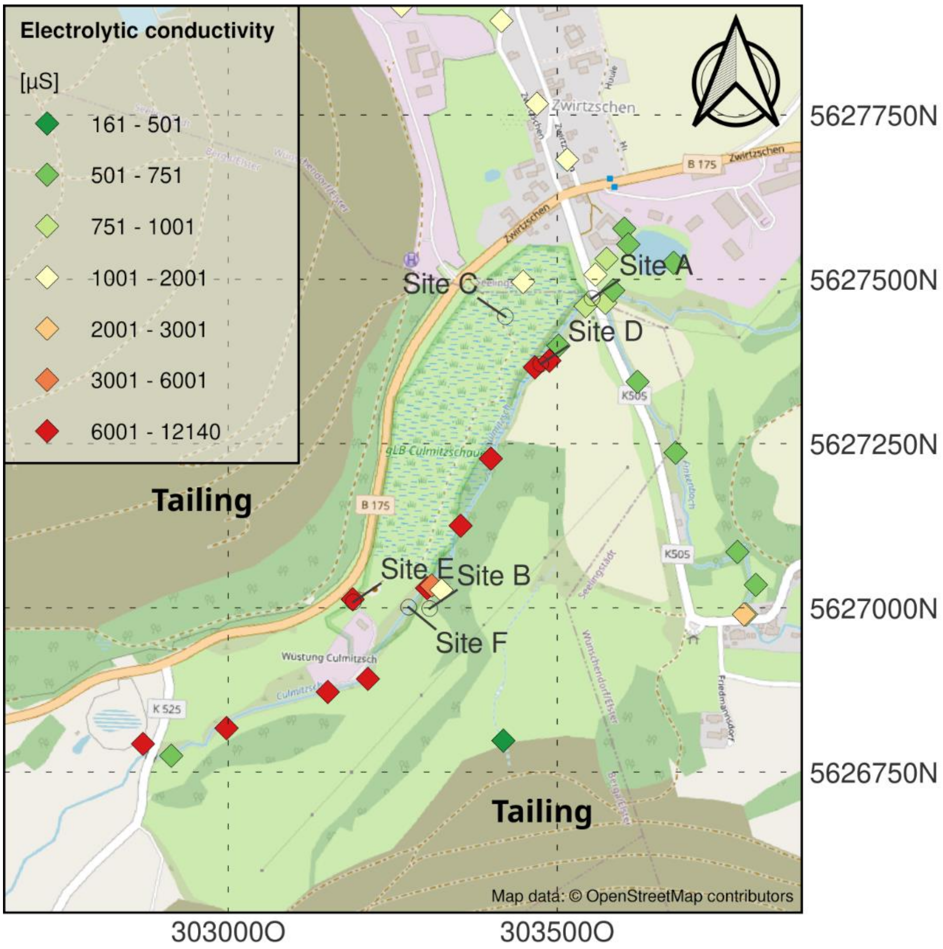

Suppl. Figure S1. Map of the sampling area with sites A (lowest influence) through F (highest contamination) along a salinity gradient resulting from seepage waters from two tailings located to the northwest and southeast.

Suppl. Table S1. Element concentrations in three independent samples were measured for soil or sediment samples.

site	Cd	Co	Cu	Fe	Mn	Ni	Pb	Sr	Zn	Cs
**soil/sediment samples** (µg/g)										
**A**	0.17 ± 0.01	0.12 ± 0.01	0.96 ± 0.00	1.60 ± 0.10	484.40 ± 3.13	6.90 ± 0.14	0.02 ± 0.00	9.90 ± 0.07	32.34 ± 0.20	0.17 ± 0.00
**B**	0.13±0.01	0.43 ± 0.00	2.34 ± 0.11	3.42 ± 0.20	194.00 ± 1.22	1.12 ± 0.03	0.48 ± 0.01	8.81 ± 0.00	3.90 ± 0.03	0.28 ± 0.01
	0.14 ± 0.01	0.44 ± 0.01	1.38 ± 0.02	2.31 ± 0.12	198.36 ± 0.30	1.30 ± 0.11	0.31 ± 0.00	7.03 ± 0.03	5.80 ± 0.22	0.11 ± 0.00
	0.14 ± 0.01	0.54 ± 0.01	1.37 ± 0.01	3.58 ± 0.20	257.00 ± 1.41	1.03 ± 0.03	0.40 ± 0.01	9.22 ± 0.01	3.60 ± 0.20	0.11 ± 0.00
**C**	0.43 ± 0.03	0.35 ± 0.01	0.66 ± 0.07	5.94 ± 0.20	31.20 ± 0.32	0.49 ± 0.07	1.31 ± 0.01	8.45 ± 0.10	2.80 ± 0.20	0.19 ± 0.00
	0.65 ± 0.01	0.29 ± 0.00	0.82 ± 0.04	11.00 ± 0.22	24.57 ± 0.22	0.64 ± 0.09	5.12 ± 0.04	13.81 ± 0.05	4.89 ± 0.20	0.24 ± 0.01
	1.43 ± 0.08	0.37 ± 0.01	0.66 ± 0.03	16.36 ± 0.08	21.80 ± 0.14	0.71 ± 0.03	4.58 ± 0.01	13.67 ± 0.20	8.07 ± 0.13	0.52 ± 0.01
**D**	0.19 ± 0.01	1.55 ± 0.03	3.46 ± 0.07	17.50 ± 0.10	210.20 ± 2.19	2.91 ± 0.06	1.17 ± 0.01	33.15 ± 0.07	22.59 ± 0.08	4.03 ± 0.00

**E**	1.08 ± 0.07	0.62 ± 0.00	3.66 ± 0.07	1.80 ± 0.10	1595.00 ± 8.25	19.60 ± 0.10	0.28 ± 0.00	23.62 ± 0.10	33.05 ± 1.00	9.35 ± 0.08
	0.97 ± 0.01	0.20 ± 0.00	4.58 ± 0.07	1.42 ± 0.07	698.00 ± 11.03	16.58 ± 0.70	0.16 ± 0.00	21.55 ± 0.03	40.27 ± 0.20	7.69 ± 0.05
	0.96 ± 0.04	0.09 ± 0.00	3.61 ± 0.05	1.40 ± 0.20	669.00 ± 14.14	15.80 ± 0.32	0.09 ± 0.00	25.01 ± 0.03	44.50 ± 0.32	7.47 ± 0.05
**F**	0.08 ± 0.00	0.58 ± 0.02	1.32 ± 0.03	7.58 ± 0.10	56.00 ± 0.85	1.55 ± 0.05	0.72 ± 0.00	11.28 ± 0.04	6.85 ± 0.05	2.04 ± 0.03
**water samples** (µg/l)										
**A**	< 0.1	0.49 ± 0.00	2.06 ± 0.01	40.00 ± 1.00	146.50 ± 0.00	3.40 ± 0.20	< 0.05	248.00 ± 3.00	12.90 ± 0.70	0.32 ± 0.01
**D**	< 0.4	0.92 ± 0.00	< 1	30.00 ± 4.00	876.40 ± 0.40	4.80 ± 0.90	< 0.1	1206.00 ± 5.00	< 20	57.20 ± 0.70
**E**	0.20 ± 0.03	1.08 ± 0.01	2.90 ± 0.20	118.00 ± 1.00	2395.00 ± 3.00	8.80 ± 0.70	0.10 ± 0.00	414.60 ± 0.10	65.00 ± 2.00	17.80 ± 0.20
**F**	0.53 ± 0.07	1.50 ± 0.10	< 1	25.00 ± 4.00	925.00 ± 3.00	22.00 ± 2.00	< 0.1	1210.00 ± 10.00	< 20	56.10 ± 0.20

	**Ba**	**Ca**	**K**	**Mg**	**Na**	**fluoride**	**chloride**	**sulfate**
**A**	0.04 ± 0.001	66.00 ± 0.30	15.60 ± 0.20	22.30 ± 0.30	65.00 ± 1.00	0.26 ± 0.01	87.80 ± 0.30	134.90 ± 0.50
**D**	0.06 ± 0.001	333.00 ± 2.00	33.50 ± 0.30	729.00 ± 4.00	1504.00 ± 9.00	1.81 ± 0.01	1037.00 ± 5.00	5535.00 ± 27.00
**E**	0.11 ± 0.001	115.29 ± 0.02	13.20 ± 0.10	126.90 ± 0.90	669.00 ± 3.00	1.03 ± 0.01	780.00 ± 1.00	781.00 ± 10.00
**F**	0.05 ± 0.001	334.40 ± 0.40	33.70 ± 0.30	733.00 ± 6.00	1517.00 ± 22.00	1.80 ± 0.01	1027.00 ± 4.00	5472.00 ± 20.00

For description of sampling sites and information on multiple sampling times, see Material and Methods; additional ions were measured in the water samples to determine water chemistry (mg/l).

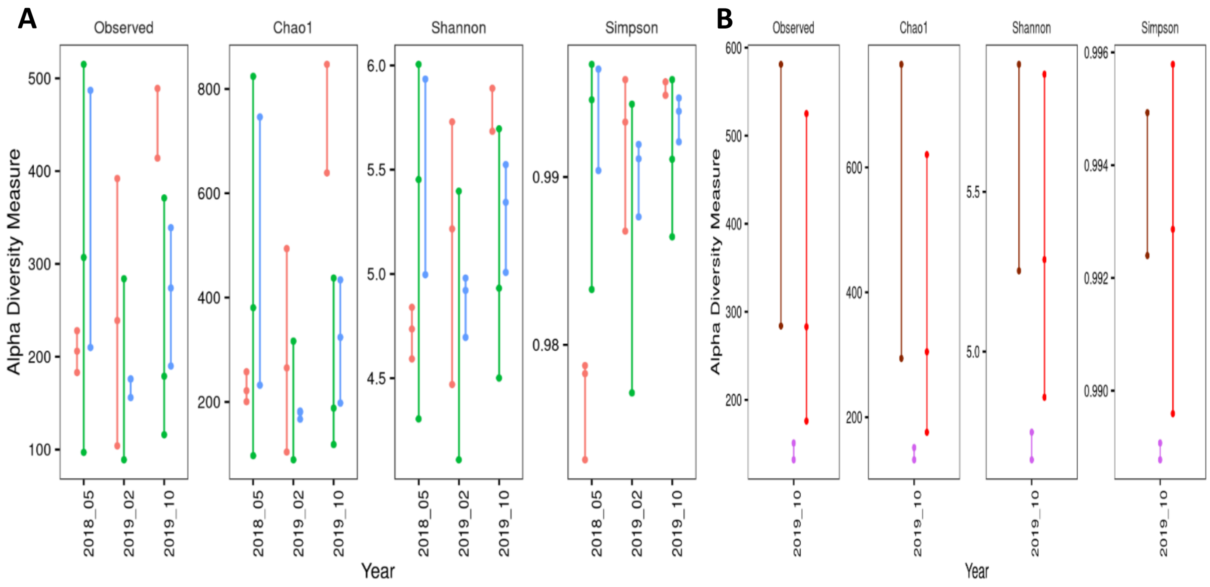

Suppl. Figure S2. Alpha-diversity measurements of the indices Chao1 as estimator for richness, Shannon for diversity and Simpson for dominance in bacterial microbiomes to compare sampling points over time. The analyses were carried out with the rarefied 16S rRNA sequences of bacterial microbiomes for soil (A) and sediment (B) samples. Red, site E; green, site B; blue, site C; brown, site F; purple, site A; light red, site D.

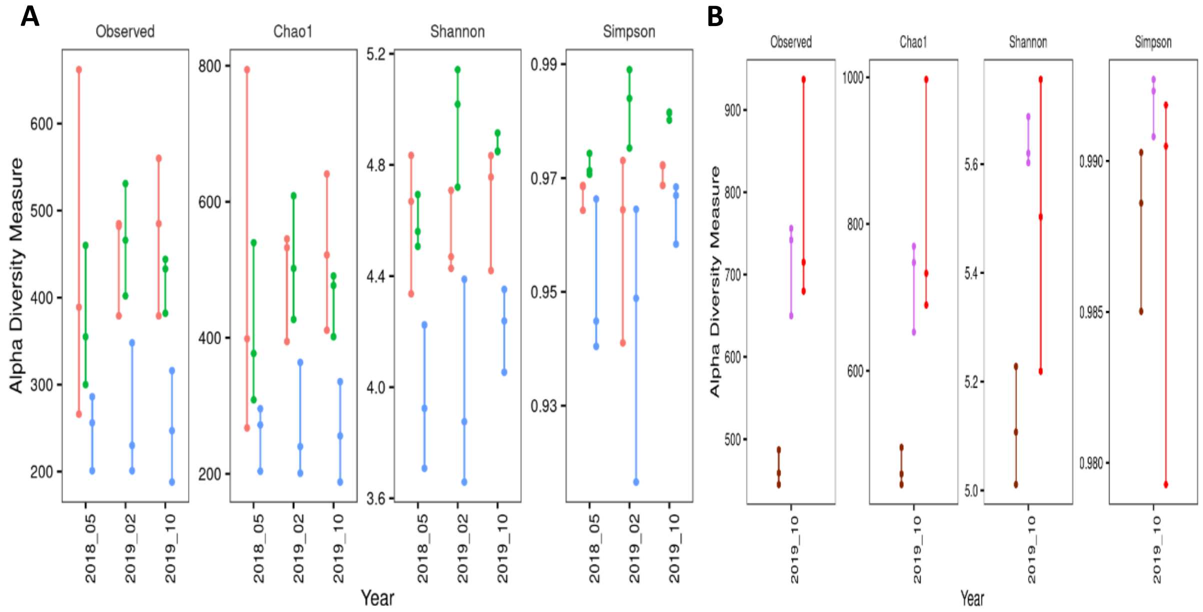

Suppl. Figure S3. Alpha-diversity measurements of the indices Chao1 as estimator for richness, Shannon for diversity and Simpson for dominance in fungal microbiomes to compare sampling points over time. The analyses were carried out with the rarefied ITS microbiomes for soil (A) and sediment (B) samples. Red, site E; green, site B; blue, site C; brown, site F; purple, site A; light red, site D.

Suppl. Table S2. Microbial count of microbes in soil/sediment (cfu/g) and water (cfu/ml) samples.

samples		isolation medium	cfu/g or cfu/ml
site A	sediment	Std I + 10% NaCl	6.76 x 10^4
		R2A + 10% NaCl	7.81 x 10^4
	water	R2A + 10% NaCl	2.78 x 10^3
site B	soil	Std I	3.02 x 10^7
		soil extract	9.37 x10^6
		Std I + 10% NaCl	5.89 x 10^4
		Std I +20% NaCl	0
		MEA	7.66 x 10^5
site c	soil	Std I	1.18 x 10^7
		soil extract	1.00 x10^7
		Std I + 10% NaCl	1.05 x 10^5
		Std I + 20% NaCl	6.67 x10^2
		MEA	1.44 x 10^6
site D	sediment	Std I + 10% NaCl	7.41 x 10^4
		R2A + 10% NaCl	4.44 x 10^4
	water	R2A + 10% NaCl	2.81 x 10^4
site E	soil	Std I	7.28 x 10^6
		soil extract	7.45 x 10^6
		Std I + 10% NaCl (10/2019)	2.47 x 10^5
		Std I + 20% NaCl	3.33 x 10^2
		MEA	5.00 x 10^4
site F	sediment	Std I + 10% NaCl	2.89 x 10^5
		R2A + 10% NaCl	2.00 x 10^5
	water	R2A + 10% NaCl	1.67 x 10^3

Suppl. Table S3. Salt tolerance of isolates grown on NaCl or Na_2SO_4 containing media.

		Number of isolates	Percentage*
**NaCl**	10%	101	74
	20%	38	28
	30%	43	32
**Na_2SO_4**	10%	116	85
	14%	107	79
	17%	93	68
	Std I	129	95

* Percentage of strains growing with salt addition of 136 tested.

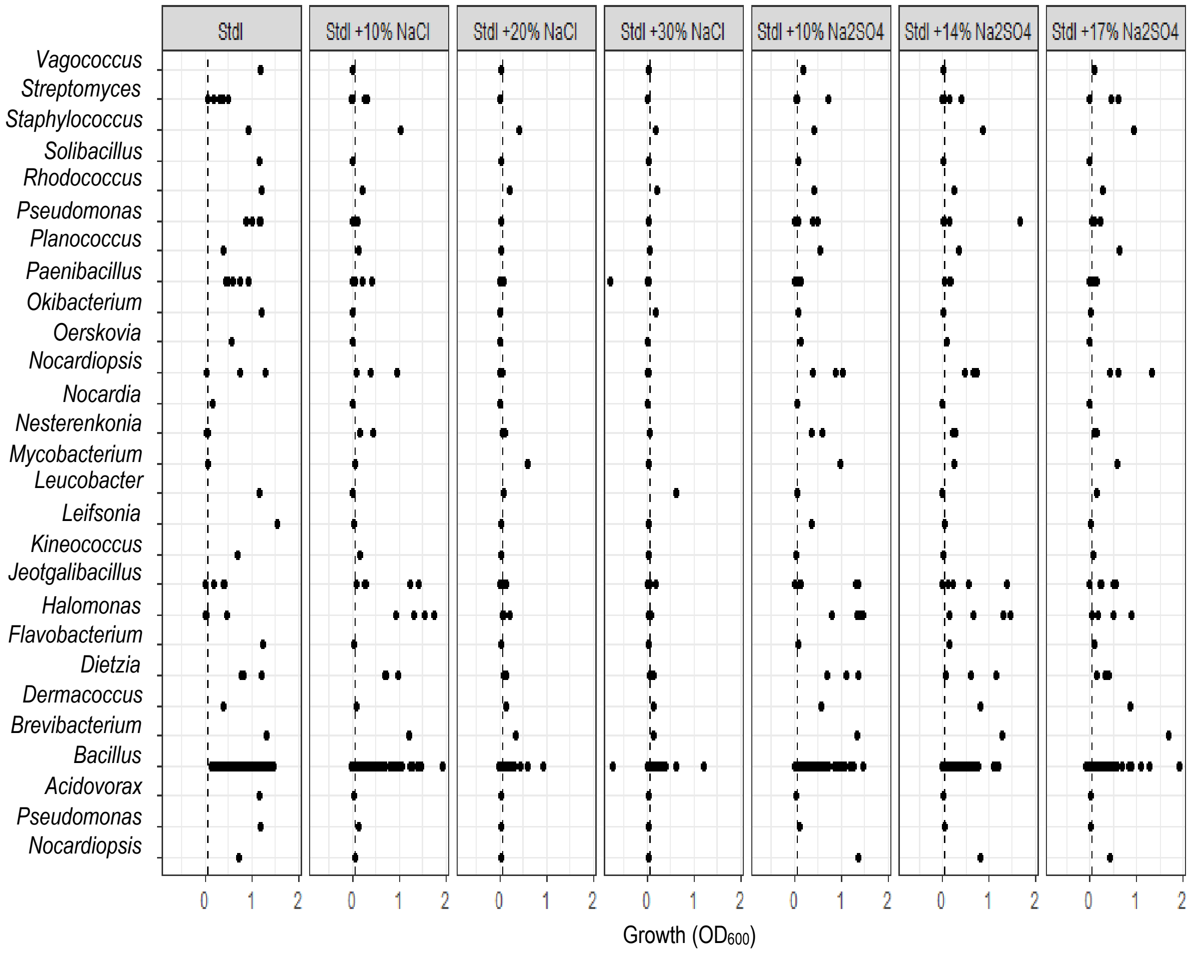

Suppl. Figure S4. Distribution of salt tolerance for genera isolated as per growth in salt-rich media. Each dot represents one isolate with the maximum tolerated salt for growth.

Suppl. Table S4. Minerals formed on different salt containing media and mineral micromorphology per isolate.

isolate		Cs_2SO_4	CsCl	$SrCl_2$
5	*Bacillus sp.*	tetrahedon	tetrahedon	-
9	*Bacillus sp.*	tetrahedon	skin	-
10	*Bacillus sp.*	skin	tetrahedon	singletons
13	*Bacillus sp.*	tetrahedon / needles	tetrahedon	singletons
16	*Bacillus sp.*	tetrahedon	tetrahedon	singletons
21	*Bacillus sp.*	tetrahedon	tetrahedon	singletons
24	*Bacillus sp.*	tetrahedon	tetrahedon	singletons
44	*Bacillus sp.*	tetrahedron	tetrahedon	singletons
49	*Bacillus sp.*	tetrahedron	tetrahedron	few singletons
51	*Bacillus sp.*	tetrahedron	tetrahedon	singletons
53B	*Bacillus sp.*	tetrahedron	tetrahedon	singletons
72	*Bacillus sp.*	tetrahedron	tetrahedon	-
78	*Bacillus sp.*	tetrahedron	tetrahedon / needles	singletons
79	*Bacillus sp.*	tetrahedron	tetrahedon	-
80.1	*Bacillus sp.*	tetrahedron	needles	singletons
85	*Bacillus sp.*	tetrahedron	tetrahedron	-
101	*Bacillus sp.*	tetrahedron	tetrahedron	-
106	*Leucobacter sp.*	tetrahedron	tetrahedon	singletons
107	*Bacillus sp.*	tetrahedon / needles	tetrahedon	singletons
130	*Bacillus sp.*	tetrahedron	tetrahedon	singletons
A3	*Bacillus sp.*	tetrahedron	tetrahedron	aggregates
A16	*Jeotgalibacillus sp.*	needles	needles	singletons
A26	*Halomonas sp.*	tetrahedron	tetrahedon	singletons
A28	*Bacillus sp.*	needles	needles	aggregates
A50	*Jeotgalibacillus sp.*	needles	needles	singletons
B15	*Bacillus sp.*	tetrahedron	tetrahedon	singletons
B16	*Rhodococcus sp.*	needles	needle aggregates	singletons
B48	*Staphylococcus sp.*	tetrahedron	tetrahedron	aggregates
B54	*Brevibacterium sp.*	tetrahedron	tetrahedon	singletons + aggregates
B59	*Dietzia sp.*	tetrahedron	tetrahedon	singletons
B61	*Planococcus sp.*	tetrahedron	tetrahedon	singletons
B75	*Dermacoccus sp.*	tetrahedron	tetrahedon	singletons
B76	*Mycobacterium sp.*	tetrahedron	tetrahedon	singletons

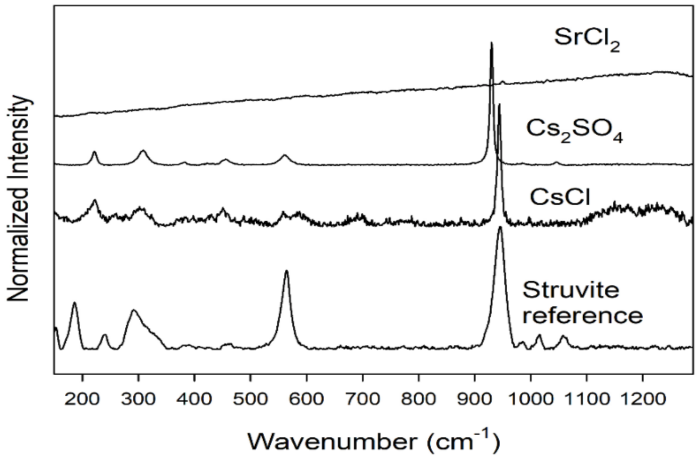

Suppl. Figure S5. Raman spectra of crystals that precipitated in experiments with CsCl, Cs_2SO_4, and $SrCl_2$ solutions in comparison to a reference spectrum of struvite (RRUFF ID: R050540). The spectrum of the $SrCl_2$–rich sample displays a faint peak at ca. 950 cm-1, which

can be assigned to a phosphate group. The spectra of Cs-rich crystals are in good agreement with the reference spectrum of struvite.

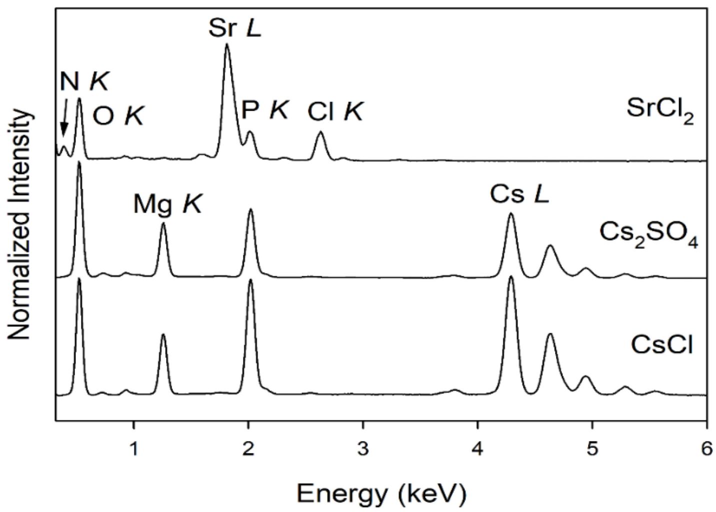

Suppl. Figure S6. Energy-dispersive X-ray (EDX) emission spectra of crystals that precipitated in experiments with CsCl, Cs_2SO_4, and $SrCl_2$ solutions. Main elements in the Cs-rich crystals are O, Mg, and P, indicating a struvite composition in which the Cs replaces the monovalent Ammonium group. The Sr-rich crystals seem to represent an assemblage of two phases: $SrCl_2$ and Sr-rich struvite, the latter containing N.
